# CT-based deep learning radiomics biomarker for programmed cell death ligand 1 expression in non-small cell lung cancer

**DOI:** 10.1186/s12880-024-01380-8

**Published:** 2024-07-31

**Authors:** Ting Xu, Xiaowen Liu, Yaxi Chen, Shuxing Wang, Changsi Jiang, Jingshan Gong

**Affiliations:** 1 The Second Clinical Medical College of Jinan University, Shenzhen, 518020 China; 2 Department of Radiology, Shenzhen People’s Hospital (The Second Clinical Medical College of Jinan University, The First Affiliated Hospital of Southern University of Science and Technology), 1F, Building 4, No. 1017 Dongmen North Road, Shenzhen, 518020 China

**Keywords:** Deep learning, Non-small cell lung cancer, Programmed cell death ligand 1, Radiomics, Immune checkpoint inhibitors

## Abstract

**Background:**

Programmed cell death ligand 1 (PD-L1), as a reliable predictive biomarker, plays an important role in guiding immunotherapy of lung cancer. To investigate the value of CT-based deep learning radiomics signature to predict PD-L1 expression in non-small cell lung cancers(NSCLCs).

**Methods:**

259 consecutive patients with pathological confirmed NSCLCs were retrospectively collected and divided into the training cohort and validation cohort according to the chronological order. The univariate and multivariate analyses were used to build the clinical model. Radiomics and deep learning features were extracted from preoperative non-contrast CT images. After feature selection, Radiomics score (Rad-score) and deep learning radiomics score (DLR-score) were calculated through a linear combination of the selected features and their coefficients. Predictive performance for PD-L1 expression was evaluated via the area under the curve (AUC) of receiver operating characteristic, the calibration curves, and the decision curve analysis.

**Results:**

The clinical model based on Cytokeratin 19 fragment and lobulated shape obtained an AUC of 0.767(95% CI: 0.673–0.860) in the training cohort and 0.604 (95% CI:0.477–0.731) in the validation cohort. 11 radiomics features and 15 deep learning features were selected by LASSO regression. AUCs of the Rad-score were 0.849 (95%CI: 0.783–0.914) and 0.717 (95%CI: 0.607–0.826) in the training cohort and validation cohort, respectively. AUCs of DLR-score were 0.938 (95%CI: 0.899–0.977) and 0.818(95%CI:0.727–0.910) in the training cohort and validation cohort, respectively. AUCs of the DLR-score were significantly higher than those of the Rad-score and the clinical model.

**Conclusion:**

The CT-based deep learning radiomics signature could achieve clinically acceptable predictive performance for PD-L1 expression, which showed potential to be a surrogate imaging biomarker or a complement of immunohistochemistry assessment.

## Background

Lung cancer is the second most commonly diagnosed cancer worldwide [1, 2]. Although early-stage detection through low-dose CT screening and mini-invasive video-assisted thoracoscopic surgery have improved patients’ survival and life quality greatly, lung cancer remains the leading cause of cancer-related death due to approximately 80% of lung cancers being diagnosed at advanced stage, which are unresectable and systemic chemotherapy is the only option [3, 4]. Only 20-40% of patients response to the standard platinum-based chemotherapy [5]. With the development of immunotherapy, the management of lung cancers has evolved enormously recently. Non-small cell lung cancer (NSCLC) accounts for about 85% in all lung cancers, including the most common subtypes such as lung adenocarcinoma and lung squamous cell carcinoma [6]. The first-generation antibody-based immunotherapy, which targets at blocking the receptor and/or ligand interactions of molecules, such as programmed cell death protein 1 (PD-1) and its ligand (PD-L1) or cytotoxic T lymphocyte antigen-4 can modulate antitumor responses, had shown remarkably response durable in NSCLCs [7]. Unfortunately, only 17-21% of patients with NSCLCs demonstrated a response to anti–PD-1 or PD-L1 therapy [8, 9]. Therefore, acknowledgment of which patients would benefit from immune checkpoint inhibitors (ICIs) is needed in NSCLCs treatment strategies. Many studies revealed that tumor mutational burden and PD-L1 expression were independent predictive factors for the response of ICIs [10, 11]. However, acknowledging these biomarkers requires invasive procedures to obtain tumor tissue specimens for gene sequencing or immunohistochemistry (IHC) staining, which are time-consuming and expensive. Furthermore, obtaining tissue specimens is difficult, even impossible in most clinical scenarios for patients with advanced NSCLC.

As a noninvasive technique, CT has been widely implemented in the diagnosis, staging, treatment planning, and response assessment of NSCLCs through radiologists’ visual interpreting, which uses only a few metrics of imaging. The development of computer science and artificial intelligent results into the emergence of radiomics, which extracts high-dimensional features from medical imaging data to decode imaging phenotype to achieve comprehensive clinical goals [12]. In recent years, convolutional neural network (CNN) with multiple network structures has been widely used in radiological tumor research. They can extract a large number of useful deep learning (DL) features for tumor grade prediction, lymph node metastasis prediction and risk prognosis prediction [13–15]. However, the construction of CNN often requires a large number of samples, and most medical studies often have a small sample size. Therefore, transfer learning is widely used in the field of medical deep learning, which can alleviate the limitation of small data sets [16]. Transfer learning involves the use of pre-trained neural networks on other images and allows existing training models to be applied to unsolved problems, thus greatly reducing the need for a large amount of training data. The purpose of this study was to develop deep learning radiomics signature as a surrogate imaging biomarker for PD-L1 expression of NSCLC, using transfer learning to extract features from CT images, in order to provide decision-making support for selecting patients who would benefit from ICIs treatment.

## Materials and methods

### Patients

**Fig. 1:**
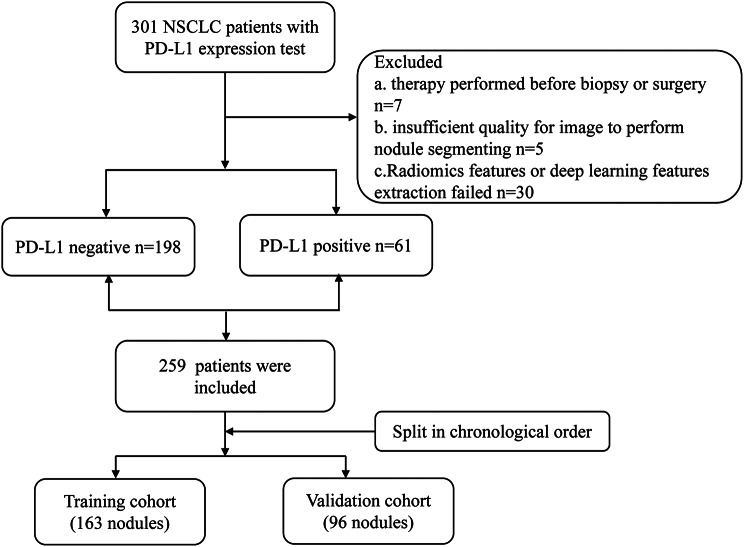
Flowchart shows inclusion and exclusion criteria for the study

This study was approved by the Local Hospital Ethics Committee of our hospital with the waiver of informed consent due to the retrospective nature. We searched our hospital’s database for patients who had pathological diagnosis of NSCLC via biopsy or surgery receiving CT examinations being performed within 3 months before biopsy or surgery and IHC staining for PD-L1 expression. Exclusion criteria were as follows: (1) insufficient image quality for nodule segmenting (*n* = 5); (2) undergoing anti-tumor therapy (radiotherapy, chemotherapy or chemoradiotherapy) before biopsy or surgery (*n* = 7); (3) Radiomics feature or deep learning feature extraction failed (*n* = 30). At first, 301 deemed eligible patients were identified. After excluding 42 patients, the final study cohort included 259 patients.who were divided into a training cohort and a validation cohort at a ratio of 7:3 according to the chronological order (Fig. 1). Patients before October 2021 were assigned to training sets(PD-L1 negative *n* = 131, PD-L1 positive *n* = 32), and patients after that wereassigned to validation sets (PD-L1 negative *n* = 67, PD-L1 positive *n* = 29). There was no significant difference in the distribution of PD-L1 expression in the training set and the validation set (*P* = 0.053), which could be used for model establishment and validation.

### CT acquisition and interpretation

Preoperative chest CT examinations were performed on CT scanners (Brilliance iCT, Philips Medical Systems). The acquisition parameters were as follows: 0.625 mm x 128 of a collimation; 120 kVp of tuber voltage; automatic exposure control (AEC) of tube current; a reconstruction slice thickness of 1.5 mm and a gap of 1 mm; field of view of 350 × 350 mm; matrix of 512 × 512.

Two experienced radiologists (2 years and 5 years chest CT interpretation experience), who were blinded to the clinical and PD-L1 expression, interpreted the thin slice CT images in the lung window setting to obtain the semantic features of nodules on the PACS (Picture Archiving and Communication Systems). In the semantic description of nodules, CT characteristics as following were included: vacuolar sign, cavity, pleural thickening, pleural indentation, hilar adenopathy, mediastinal adenopathy, vessel convergence, location, lobulated shape, spiculation, airbronchial sign, types of nodules and size.

### Feature extraction, feature selection and signature construction

The thin slice images in Digital Imaging and Communication in Medicine (DICOM) format derived from PACS were transferred to ITK-SNAP 3.8.0 (http://www.itksnap.org) of a personal computer for tumor segmentation and radiomics feature extraction. The regions of interest (ROIs) of tumor were manually drew using ITK-SNAP software on each thin slice to convert a three-dimensional volume of interest (3D-VOIs). Two radiologists with 2 years and 5 years of experience in thoracic CT interpretation segmented tumors in 30 randomly selected patients independently. The radiologist with 2 years of experience segmented all the nodules manually. 1834 radiomics features were extracted from the 3D-VOIs using the open-source software package Pyradiomics (https://github.com/Radiomics/pyradiomics).

A pre-trained CNN, ResNet 50, was used for transfer learning to extract deep learning features from thin CT images of NSCLCs. First, the image with the largest tumor area per patient was selected and the grayscale values were normalized into the range [− 1,1] using a min-max transformation. Then each cropped subregion image was resized to 224 × 224, and the resulting image was used as model input [17, 18].

**Fig. 2:**
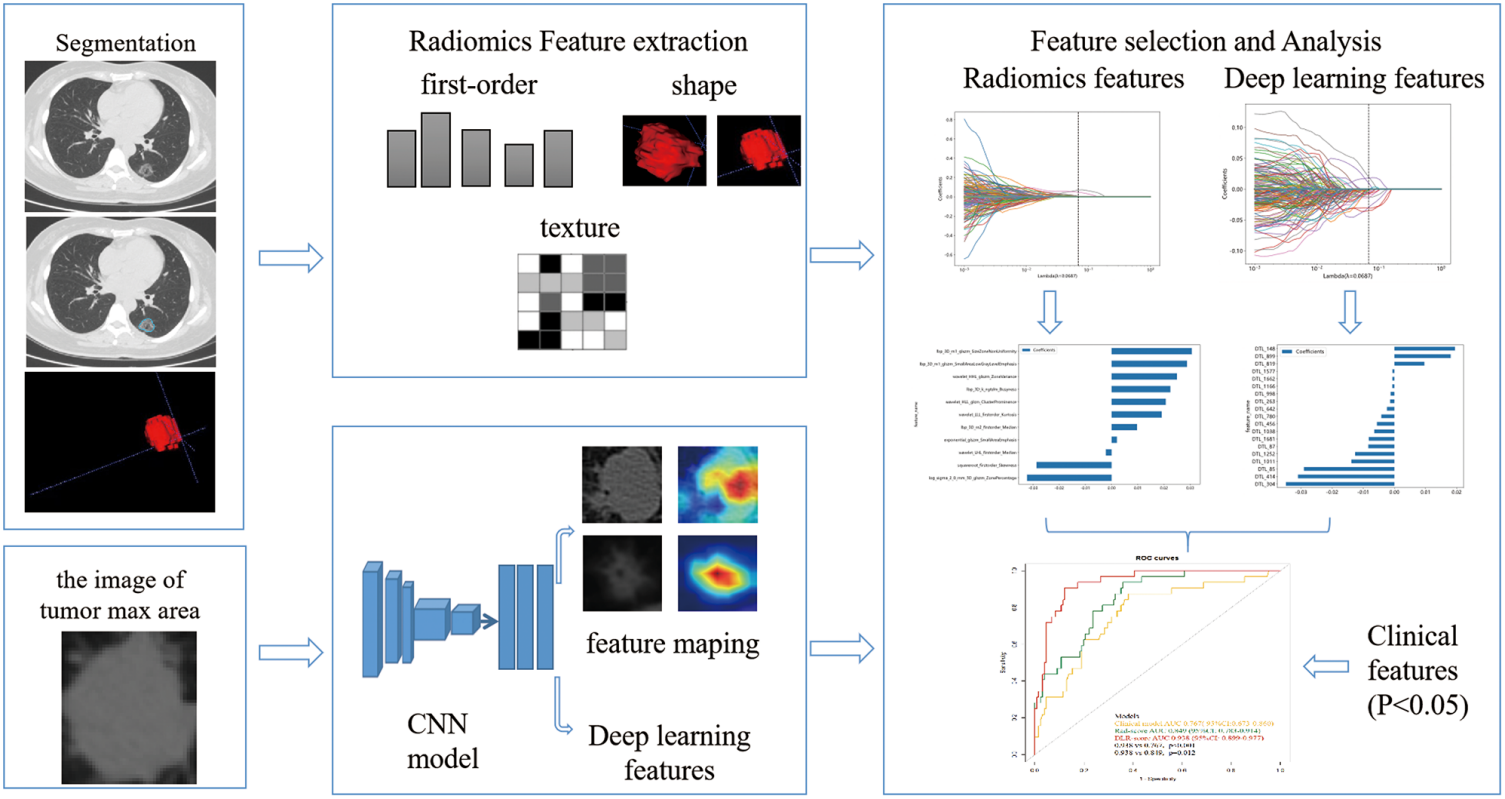
The models workflow

Z-Score normalization was implemented to reduce the influence of features’ scales. Intraclass correlation coefficients (ICC) of interobservers were implemented to roll out those radiomics or deep learning features with low repeatability (ICC ≤ 0.75). Pearson correlation was performed to exclude the radiomics or deep learning features with high correlations (*r* > 0.90). Then the retained features were introduced into the least absolute shrinkage and selection operator (LASSO) regression with 5-fold cross-validation to select radiomics or deep learning features which were strong association with PD-L1. Radiomics score (Rad-score) and deep learning radiomics score (DLR-score) for each patient were calculated through a linear combination of the selected features weighted by their coefficient. Feature selection procedure was implemented on both radiomics features and deep learning features in the training cohort. The signatures trained on the training cohort were applied to the validation cohort for testing in independent cases. The workflow of model building is shown in Fig. 2.

### PD-L1 testing

PD-L1 expression of NSCLCs was assessed using immunohistochemical staining and reported as tumor proportion score (TPS). TPS is defined as the percentage of tumor cells stained with PD-L1 membrane of any intensity. The PD-L1 expression was dichotomized according to TPS level (TPS < 1% is negative, TPS ≥ 1% is positive). Finally, there were 198 PD-L1 negative cases and 61 PDL1 positive cases.

### Statistical analysis and Model Development

For clinical metrics, univariate analysis (t-test, Mann-Whitney U rank test, χ^2^ test, or Fisher’s precise probability test) was used to select those which were related to PD-L1 expression, and then introduced into a multivariate logistic regression model. The predictive performances of the clinical model, the Rad-score and the DLR-score were assessed by area under the curve (AUC) of receiver operating characteristic (ROC) which were compared using the DeLong method. The calibration effectiveness, the goodness of fit, the net benefit and the clinical effectiveness of the better model were evaluated using the calibration curve, the Hosmer-Lemeshow and the decision curve. ROC analysis of models was performed to obtain the optimal cut-off value [19, 20].

## Results

### Patient characteristics and clinical model

Demographic characteristics of all patients were showed in Table 1. At univariable analysis, Cytokeratin 19 fragment (*P* = 0.047) and lobulated shape(*P* = 0.014) were related to PD-L1 with statistical significance (Table 2) in training cohort. Multivariate logistic regression revealed that Cytokeratin 19 fragment and lobulated shape were independent risk factors for PD-L1 (Table 2). A clinical model based on these two clinical features yielded predictive performance with an AUC of 0.767(95% CI: 0.673–0.860) for PD-L1 in the training cohort and 0.604 (95% CI:0.477–0.731) in validation cohort.

**Table 1:** Demographic and CT characteristics of all patients

variables	Training set(*n* = 163)				Validation set (*n* = 96)					
	All	PD-L1 negative(*n* = 131)	PD-L1positive(*n* = 32)	*P* Value		All	PD-L1 negative(*n* = 67)	PD-L1 positive(*n* = 29)		*P* Value
Age	56.90 ± 12.09	56.53 ± 12.44	58.41 ± 10.57	0.432		58.60 ± 14.46	57.81 ± 14.61	60.45 ± 14.19		0.414
CK_19	2.25(1.74,3.06)	2.11(1.68,2.92)	2.79(2.08,4.18)	0.001		2.57(1.98,3.77)	2.57(1.63,3.58)	2.56(2.16,4.19)		0.160
CEA	1.72(0.97,2.84)	1.55(0.92,2.53)	2.80(1.66,7.04)	< 0.001		2.13(1.30,3.83)	1.84(1.23,3.43)	3.23(1.75,6.60)		0.100
NSE	10.90(9.14,12.70)	10.80(8.98,12.50)	11.40(10.12,13.58)	0.090		11.30(9.54,13.48)	11.85(9.46,14.00)	10.90(9.55,12.30)		0.357
Gender				0.204						0.827
female	100(61.35%)	84(64.12%)	16(50.00%)			53(55.21%)	36(53.73%)	17(58.62%)		
male	63(38.65%)	47(35.88%)	16(50.00%)			43(44.79%)	31(46.27%)	12(41.38%)		
Smoking				1						1
absence	129(79.14%)	104(79.39%)	25(78.12%)			80(83.33%)	56(83.58%)	24(82.76%)		
presence	34(20.86%)	27(20.61%)	7(21.88%)			16(16.67%)	11(16.42%)	5(17.24%)		
Family history				0.931						0.115
no cancer	133(81.60%)	107(81.68%)	26(81.25%)			87(90.62%)	63(94.03%)	24(82.76%)		
lung cancer	12(7.36%)	10(7.63%)	2(6.25%)			4(4.17%)	1(1.49%)	3(10.34%)		
other cancers	18(11.04%)	14(10.69%)	4(12.50%)			5(5.21%)	3(4.48%)	2(6.90%)		
TNM				0.630						0.140
I	77(47.24%)	60(45.80%)	17(53.12%)			47(48.96%)	31(46.27%)	16(55.17%)		
II	7(4.29%)	5(3.82%)	2(6.25%)			2(2.08%)	0(0.00%)	2(6.90%)		
III	7(4.29%)	7(5.34%)	0(0.00%)			5(5.21%)	3(4.48%)	2(6.90%)		
IV	9(5.52%)	7(5.34%)	2(6.25%)			3(3.12%)	2(2.99%)	1(3.45%)		
unknown	63(38.65%)	52(39.69%)	11(34.38%)			39(40.62%)	31(46.27%)	8(27.59%)		
Vacuolar sign				0.677						0.247
absence	134(82.21%)	109(83.21%)	25(78.12%)			69(71.88%)	51(76.12%)	18(62.07%)		
presence	29(17.79%)	22(16.79%)	7(21.88%)			27(28.12%)	16(23.88%)	11(37.93%)		
Cavity				1.000						0.461
absence	157(96.32%)	126(96.18%)	31(96.88%)			88(91.67%)	60(89.55%)	28(96.55%)		
presence	6(3.68%)	5(3.82%)	1(3.12%)			8(8.33%)	7(10.45%)	1(3.45%)		
Pleural thickening				1.000						1
absence	109(66.87%)	88(67.18%)	21(65.62%)			47(48.96%)	33(49.25%)	14(48.28%)		
presence	54(33.13%)	43(32.82%)	11(34.38%)			49(51.04%)	34(50.75%)	15(51.72%)		
Pleural indentation				0.378						0.221
absence	75(46.01%)	63(48.09%)	12(37.50%)			37(38.54%)	29(43.28%)	8(27.59%)		
presence	88(53.99%)	68(51.91%)	20(62.50%)			59(61.46%)	38(56.72%)	21(72.41%)		
Hilar adenopathy				1.000						0.312
absence	150(92.02%)	121(92.37%)	29(90.62%)			91(94.79%)	62(92.54%)	29(100.00%)		
presence	13(7.98%)	10(7.63%)	3(9.38%)			5(5.21%)	5(7.46%)	0		
Mediastinal adenopathy				0.705						0.123
absence	148(90.80%)	120(91.60%)	28(87.50%)			88(91.67%)	59(88.06%)	29(100.00%)		
presence	15(9.20%)	11(8.40%)	4(12.50%)			8(8.33%)	8(11.94%)	0(0.00%)		
Lobulated_shape				0.180						0.427
absence	60(36.81%)	52(39.69%)	8(25.00%)			17(17.71%)	10(14.93%)	7(24.14%)		
presence	103(63.19%)	79(60.31%)	24(75.00%)			79(82.29%)	57(85.07%)	22(75.86%)		
Vessel_convergence				0.885						0.491
absence	72(44.17%)	57(43.51%)	15(46.88%)			30(31.25%)	19(28.36%)	11(37.93%)		
presence	91(55.83%)	74(56.49%)	17(53.12%)			66(68.75%)	48(71.64%)	18(62.07%)		
Spiculation				1.000						0.019
absence	77(47.24%)	62(47.33%)	15(46.88%)			35(36.46%)	30(44.78%)	5(17.24%)		
presence	86(52.76%)	69(52.67%)	17(53.12%)			61(63.54%)	37(55.22%)	24(82.76%)		
Airbronchial_sign				0.914						0.327
absence	131(80.37%)	106(80.92%)	25(78.12%)			74(77.08%)	54(80.60%)	20(68.97%)		
presence	32(19.63%)	25(19.08%)	7(21.88%)			22(22.92%)	13(19.40%)	9(31.03%)		
Types of nodule				0.225						0.914
ground-glass nodule	58(35.58%)	48(36.64%)	10(31.25%)			34(35.42%)	23(34.33%)	11(37.93%)		
part-solid ground-glass nodule	54(33.13%)	46(35.11%)	8(25.00%)			26(27.08%)	18(26.87%)	8(27.59%)		
solid nodule	51(31.29%)	37(28.24%)	14(43.75%)			36(37.50%)	26(38.81%)	10(34.48%)		
Size	12.00(8.50,18.25)	11.50(8.25,17.00)	16.00(11.13,24.31)	0.002		13.63(10.75,19.69)	13.00(9.25,18.50)	15.50(11.38,22.63)		0.139

†CEA, carcinoembryonic antigen; NSE, neuron specific enolase

**Table 2:** The univariate and multivariate logistic regression of PD-L1 expression based on clinical and CT characteristics in training cohort

variables	Univariate analysis			Multivariate logistic regression		
	OR value	95%CI	*P* value	OR value	95%CI	*P* value
Gender	2.487	0.725-8.600	0.144			
Age	0.994	0.947–1.042	0.803			
Cytokeratin 19 fragment	1.402	1.036–1.983	0.047	1.511	1.180–2.085	0.006
CEA	1.000	0.985–1.014	0.950			
NSE	1.096	0.933–1.285	0.266			
Smoking	0.543	0.137–2.346	0.426			
Famaily history	0.725	0.312–1.493	0.410			
TNM stage	0.709	0.221–1.876	0.514			
Vacuolar sign	0.744	0.190–2.628	0.655			
Cavity	0.409	0.147–4.672	0.517			
Pleural thickening	0.538	0.155–1.702	0.305			
Pleural indentation	1.601	0.464–5.695	0.457			
Hilar adenopathy	0.616	0.041–6.237	0.697			
Mediastinal adenopathy	2.890	0.327–29.307	0.344			
Vessel convergence	1.771	0.571–5.805	0.328			
Location	1.866	0.550–7.601	0.343			
Lobulated shape	4.356	1.479–16.163	0.014	5.409	1.927–19.443	0.003
Spiculation	1.122	0.310–4.138	0.860			
Airbronchial sign	2.280	0.695–7.494	0.170			
Types of nodule	1.630	0.784–3.482	0.194			
Size	1.018	0.946–1.010	0.638			

†PD-L1, programmed death ligand 1; CEA, carcinoembryonic antigen; NSE, neuron specific enolase

### Radiomics score and deep learning Radiomics score building and evaluation

**Fig. 3:**
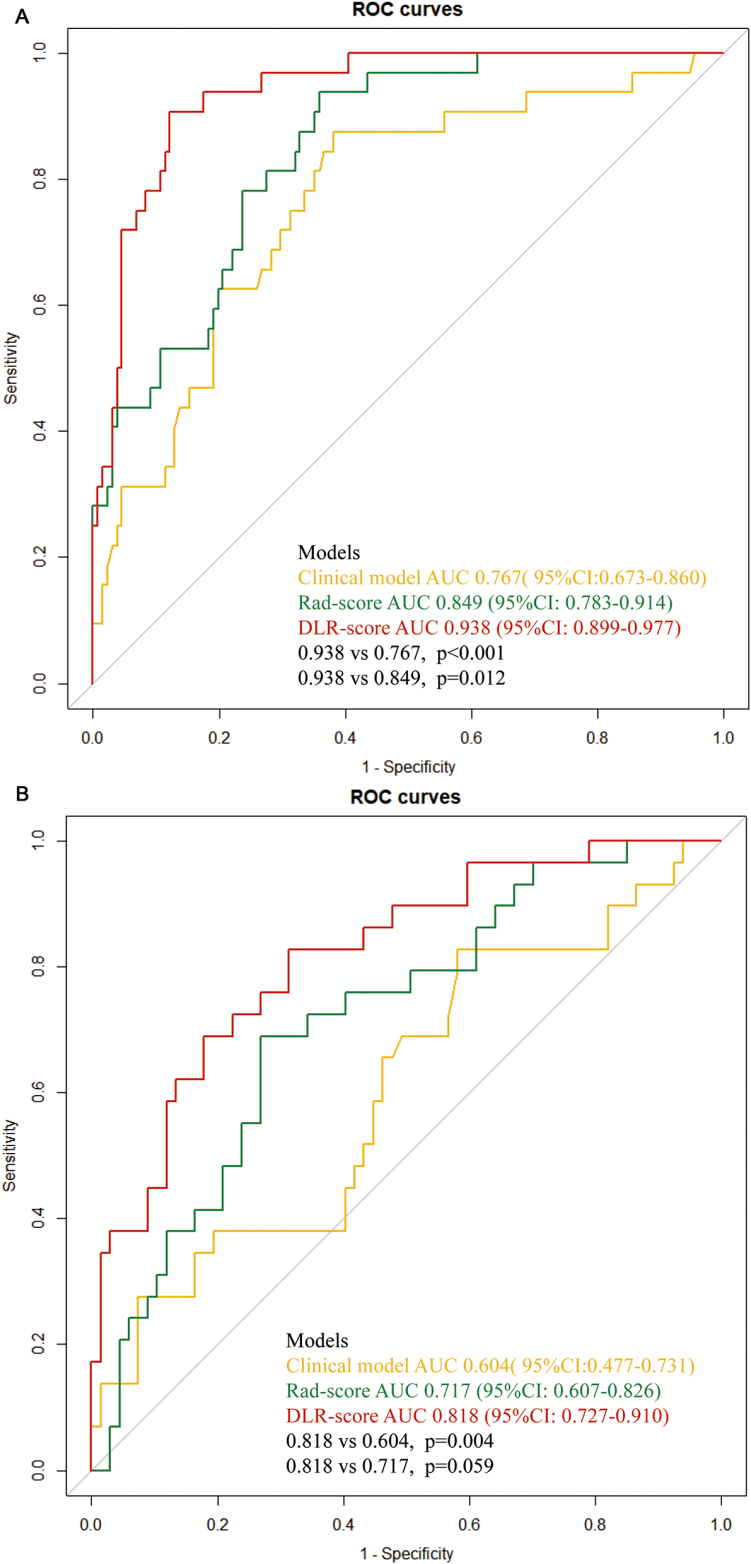
The ROC curves of clinical model, Radscore, and DLR-score in training cohort (**A**) and validation cohort (**B**), respectively

**Fig. 4:**
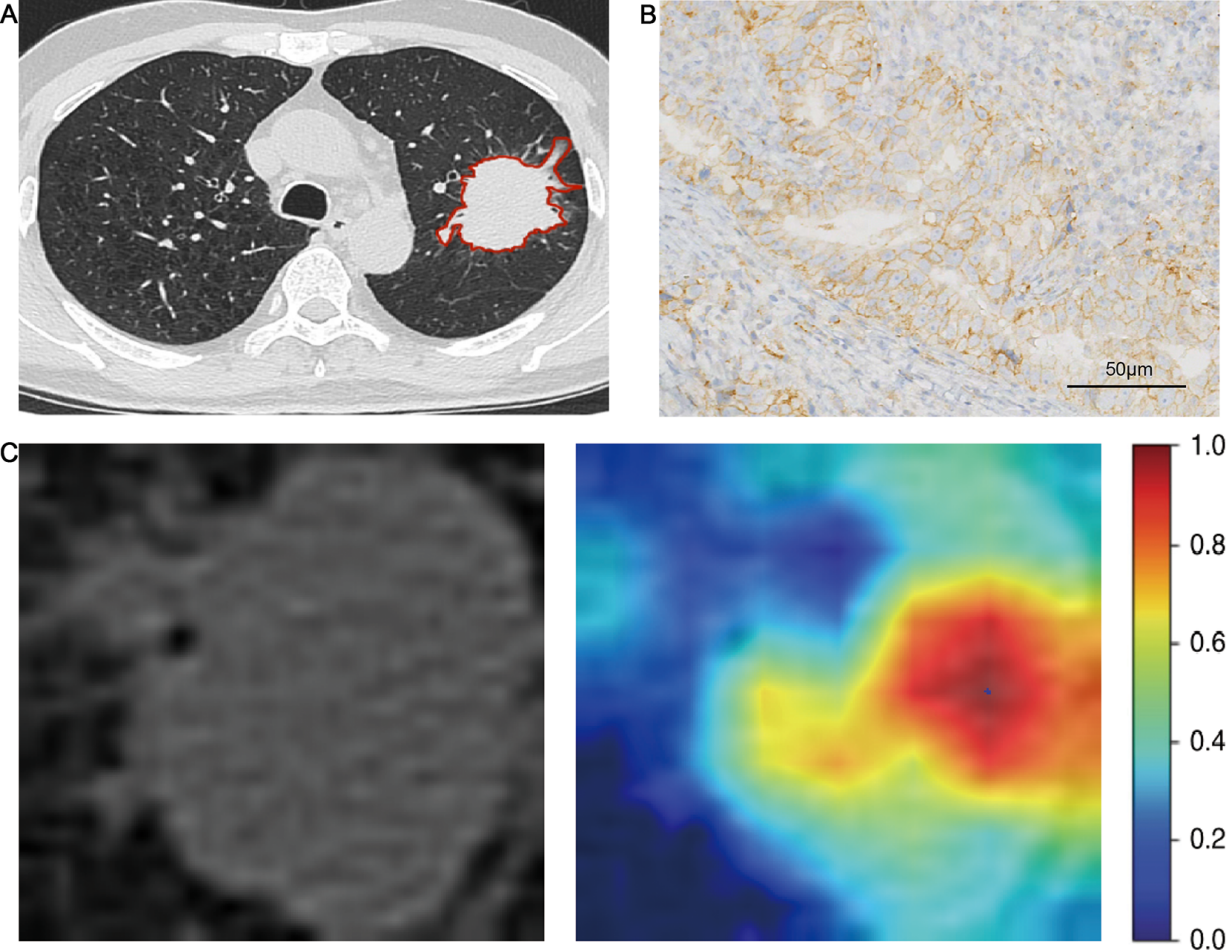
A 51-year-old man with NSCLC. (**A**) Axial CT image shows a solid nodule of the left upper lobe. DLR-score 0.839. (**B**) The Photomicrograph shows a positive expression of PD-L1(IHC; x400). (**C**) Grad-CAM visualization. Grad-CAM, gradient-weighted class activation mapping

**Fig. 5:**
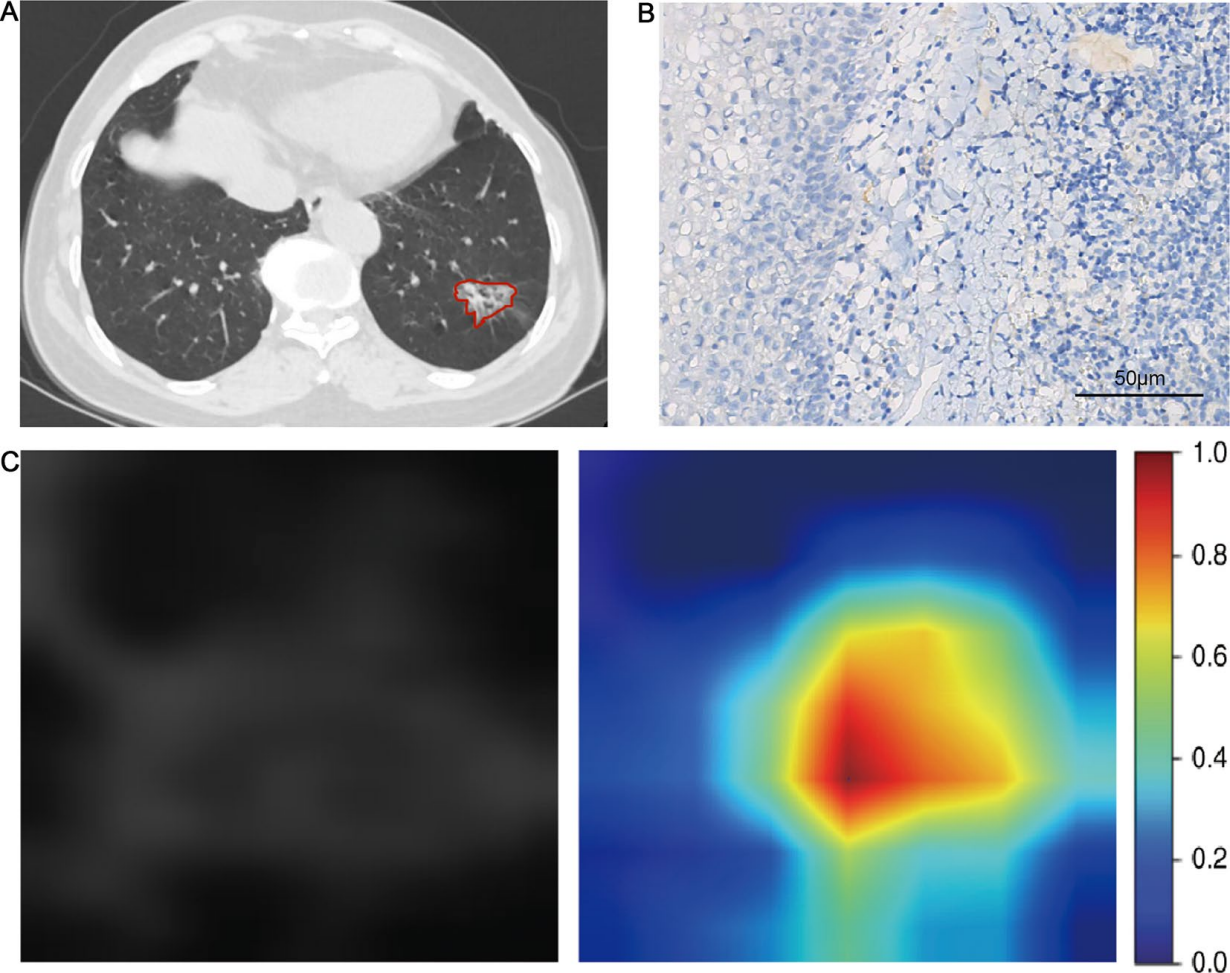
A 78-year-old man with NSCLC. (**A**) Axial CT image shows a ground glass nodule of the left lower lobe, DLR-score 0.141. (**B**) The Photomicrograph shows a negative expression of PD-L1(IHC; x400). (**C**) Grad-CAM visualization. Grad-CAM, gradient-weighted class activation mapping

**Fig. 6:**
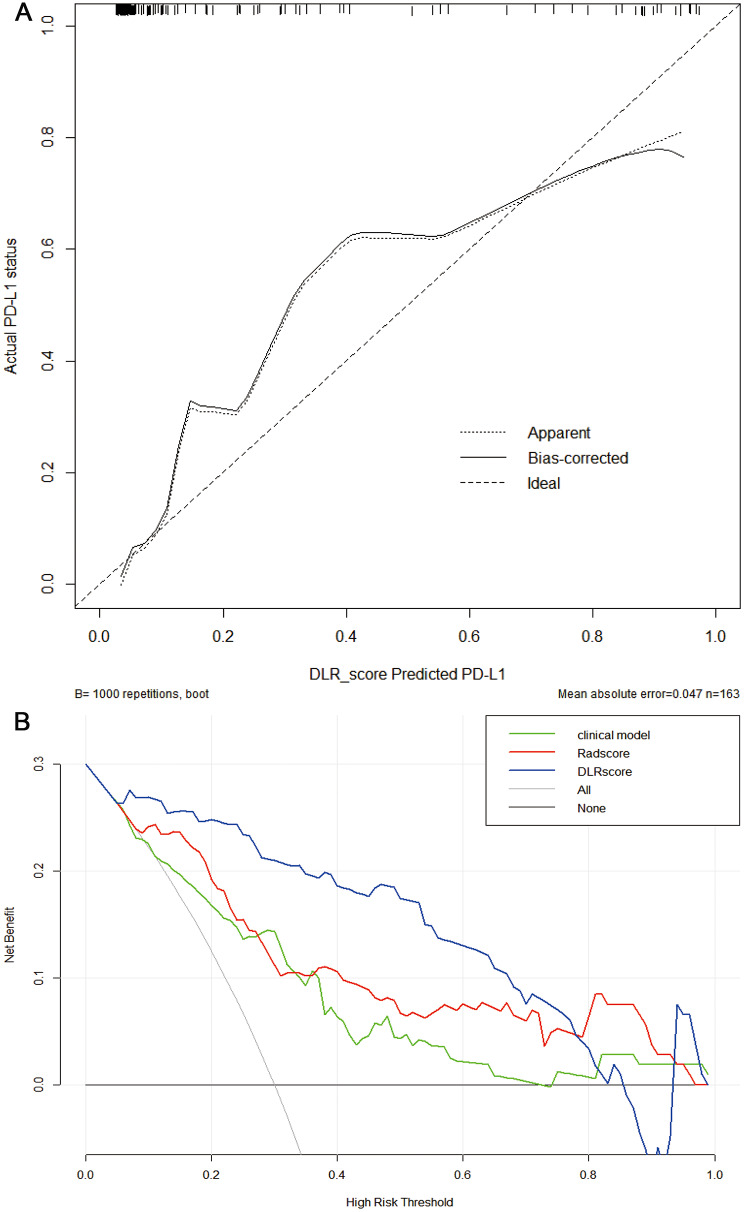
The calibration curves of the DLR-score in the training cohort (**A**). The decision curve analyses of the three models in training cohort (**B**). The DLR-score can achieve a greater net effect than the other two models

After ICC and pearson analysis, 205 radiomics features were introduced into the LASSO model. 11 features were selected to establish the Rad-score as [21]: Rad-score = 0.19631901840490795 + 0.002025*exponential_glszm_SmallAreaEmphasis + 0.022456*lbp_3D_k_ngtdm_Busyness + 0.030649*lbp_3D_m1_glszm_SizeZoneNonUniformity + 0.028801*lbp_3D_m1_glszm_SmallAreaLowGrayLevelEmphasis + 0.009714*lbp_3D_m2_firstorder_Median-0.032295*log_sigma_2_0_mm_3D_glszm_Zone%-0.028661*squareroot_firstorder_Skewness + 0.024971*wavelet_HHL_glszm_ZoneVariance + 0.020694*wavelet_HLL_glcm_ClusterProminence-0.002185*wavelet_LHL_firstorder_Median + 0.019159 * wavelet_LLL_firstorder_Kurtosis. In deep learning, 2048 DL radiomics features were extracted. After ICC and Pearson analysis, 7 features were found to be excluded. Then LASSO regression revealed that 19 DL features were strongly associated with PD-L1. Following a rule of thumb, we have retained the 15 characteristics that are most relevant to PD-L1 [22]. The DLR-score was calculated as [17]: DLR-score = 0.19631901840490795- 0.029073 * DL_85 -0.008362 * DL_87 + 0.019473 * DL_148 -0.001373 * DL_263 -0.034845 * DL_304 -0.030973 * DL_414 -0.005602 * DL_456 -0.002396 * DL_642 -0.004188 * DL_780 + 0.009646 * DL_819 + 0.018088 * DL_899 -0.013829 * DL_1011 -0.006451 * DL_1038-0.012642 * DL_1252 -0.008234 * DL_1681. AUCs of Rad-score for PD-L1 expression were 0.849 (95%CI: 0.783–0.914) and 0.717 (95%CI: 0.607–0.826) in the training cohort and validation cohort, respectively. AUCs of DLR-score for PD-L1 expression were 0.938(95%CI: 0.899–0.977) and 0.818(95%CI:0.727–0.910) in the training cohort and validation cohort, respectively. The predictive performance of the DLR-score was significantly higher than that of the clinical model and Rad-score in both cohorts (Fig. 3). The results of the ROC analysis of each model are shown in Table 3. The Youden index of the ROC curve determined the optimal cut-off value. The optimal cut-off value derived from DLR-score was 0.246, with sensitivity, specificity, PPV, NPV and accuracy, of 90.6%, 87.8%, 64.4%, 97.5%, and 88.3%, respectively. The comparison of clinical and CT features between high (more than cut-off value) and low ( less than cut-off value) DLR-score was shown in Table 4. Intending to examine the interpretability of DL features, we also visualize the network by applying a gradient-weighted class activation mapping that can provide a rough positioning map to highlight important areas of the classification target (Figs. 4 and 5). The calibration curves of the radiomics model showed a good calibration effect on the predictive efficacy of PD-L1 expression in the training cohort (Fig. 6A), and the Hosmer-Lemeshow test showed nonsignificant statistic in the training cohort (*P* = 0.141), indicating that there was no significant difference between prediction and pathology result. The decision curve analysis shows that radiomics model achieved a high net benefit at most probability thresholds, indicating that DLR-score could achieve excellent clinical effectiveness when the probability of threshold is approximately between 0% and about 80% in the training cohort (Fig. 6B).

**Table 3:** The performance of deep transfer learning radiomics signature to predict PD-L1 expression

models	cohort	AUC	*P* value*	cut-off value	sensitivity	specificity	PPV	NPV	accuracy
Clinical model	training	0.767 ( 0.673–0.860)	< 0.001	-1.431	87.5%	61.8%	35.9%	95.3%	66.9%
	validation	0.604 (0.477–0.731)	0.004	-1.431	82.8%	38.8%	36.9%	83.9%	52.1%
Rad-score	training	0.849 (0.783–0.914)	0.012	0.125	93.8%	64.1%	39.0%	97.7%	69.9%
	validation	0.717 (0.607–0.826)	0.059	0.125	75.9%	59.7%	44.9%	85.1%	64.6%
DLR-score	training	0.938 (0.899–0.977)	-	0.246	90.6%	87.8%	64.4%	97.5%	88.3%
	validation	0.818 (0.727–0.910)	-	0.246	62.1%	86.6%	66.7%	84.1%	79.2%

*The P value of the clinical model and Rad-score were obtained by performing DeLong test in two cohorts with reference to the AUC of DLR-score respectively

PPV: Positive predictive value; NPV: Negative predictive value

**Table 4:** The comparison of clinical and CT features between high DLRad-score and low DLRad-score

variables	DLRadscore		*P* value
	Low (*n* = 187)	High (*n* = 72)	
Age	57.06 ± 13.38	58.74 ± 12.01	0.355
Gender			0.034
female	118(77.1%)	35(22.9%)	
male	69(65.1%)	37(34.9%)	
Smoking			0.073
absence	156 (74.6%)	53(25.4%)	
presence	31 (62.0%)	19(38.0%)	
Family history			0.684
no cancer	161(73.2%)	59(26.8%)	
lung cancer	11(68.8%)	5(31.3%)	
other cancers	15(65.2%)	8(34.8%)	
Cytokeratin 19 fragment	2.33(1.71,3.07)	2.52(1.85,3.79)	0.039
CEA	1.72(1.00,2.84)	2.57(1.36,3.98)	0.190
NSE	10.90(9.21,12.80)	11.30(9.94,13.35)	0.098
TNM stage			0.253
I	91(73.4%)	33(26.6%)	
II	9(77.8%)	0(0.0%)	
III	10(83.3%)	2(16.7%)	
IV	8(66.7%)	4(33.0%)	
unknown	69(67.6%)	33(32.4%)	
Vacuolar sign			0.597
absence	145(71.4%)	58(28.6%)	
presence	42(75.0%)	14(25.0%)	
Cavity			0.027
absence	181(73.9%)	64(26.1%)	
presence	6(42.9%)	8(57.1%)	
Pleural thickening			0.037
absence	120(76.9%)	36(23.1%)	
presence	67(65.0%)	36(35.0%)	
Pleural indentation			0.011
absence	90(80.4%)	22(19.6%)	
presence	97(66.0%)	50(34.0%)	
Hilar adenopathy			0.006
absence	179(74.3%)	62(25.7%)	
presence	8(44.4%)	10(55.6%)	
Mediastinal adenopathy			0.001
absence	177(75.0%)	59(25.0%)	
presence	10(43.5%)	13(56.5%)	
Vessel_convergence			0.920
absence	74(72.5%)	28(27.5%)	
presence	113(72.0%)	44(28.0%)	
Lobulated_shape			0.001
absence	67(87.0%)	10(13.0%)	
presence	120(65.9%)	62(34.1%)	
Spiculation			0.001
absence	93(83.0%)	19(17.0%)	
presence	94(63.9%)	53(36.1%)	
Airbronchial sign			0.089
absence	153(74.6%)	52(25.4%)	
presence	34(63.0%)	20(37.0%)	
Types of nodule			0.003
ground-glass nodule	73(79.3%)	19(20.7%)	
part-solid ground-glass nodule	67(83.8%)	17(21.5%)	
solid nodule	51(58.6%)	36(41.4%)	
Size	11.75(8.75,17.25)	15.750(11.00,21.50)	0.001

## Discussion

In this study, we probed whether deep learning signature derived from preoperative CT could be used to predict PD-L1 expression. We developed and validated three models, clinical, radiomics, and DLR-score, for predicting PD-L1 expression by quantitative analysis of CT images of NSCLCs. In the training and validation cohorts, the DLR-score showed the best predictive performance compared to other models. The AUCs of DLR-score were 0.938(95%CI: 0.899–0.977) in the training cohort and 0.818(95%CI:0.727–0.910) in the validation cohort. DCA showed that the DLR-score can improve the predictive performance of PD-L1 expression. The high predictive performance of the DLR-score showed the possibility to be a noninvasive surrogate biomarker for PD-L1 facilitating the selection of patients who would benefit from ICIs treatment.

PD-L1 plays an important role in guiding immunotherapy of lung cancer. When the PD-L1 of tumor cells binds to the PD-1 on the surface of immune cells, a negative immune response will occur, resulting in the escape of tumor cells and promoting the occurrence, development and metastasis of tumors [23]. PD-1/PD-L1 inhibitors kill tumor cells by blocking the binding of the PD-1/PD-L1 pathway, relieving the negative regulation of immune cells and preventing immune escape [24]. PD-1/PD-L1 inhibitors were included in the first category of recommendations in NSCLC’s NCCN guidelines [25, 26]. When stratified analysis of PD-L1 in tumor tissue, it was found that PD-1/PD-L1 inhibitor was more effective in patients with PD-L1 positive [27–29]. Therefore, there is an urgent need for screening patients who might be most likely to benefit from ICIs treatment. Recent studies showed that PD-L1 expression, tumor mutational burden and tumor immune microenvironment can be used as predictive biomarkers to predict the response of ICIs treatment, but these require not only invasive procedures to obtain tissue specimens, but also time-consuming and expensive laboratory tests [7, 30]. Therefore, these biomarkers are not broadly available in clinical scenarios, especially in patients with advanced NSCLCs for tissue specimens are difficult to obtain sometimes, even impossible. Furthermore, malignancies are heterogenetic, and tissue specimens, especially those through biopsy, may harbor sample errors. Therefore, clinicians still confront the challenge to choose suitable patients for ICIs treatment.

Radiomics is a data-driven discipline based on widely available imaging data that can be used to improve diagnosis, prognosis, and clinical decision support [31]. In order to provide decision support of ICIs treatment, several studies have investigated the association between radiomics signature with PD-L1 expression and tumor immune microenvironment in several kinds of solid tumors [32–34]. Regarding PD-L1 expression in NSCLC, Jiang et al [35] derived radiomics signatures from CT, PET, and PET/CT, which achieved predictive performance to identify PD-L1 expression over 1% with AUC of 0.86, 0.62, and 0.85, respectively. Using the same algorithm, Sun et al [36] reported preoperative CT-derived radiomics signature obtained AUCs of 0.786 and 0.807 in the training and validation cohort, respectively. When combined with clinicopathological features, the predictive performance increased to 0.829 and 0.848, respectively. However, the authors did not evaluate whether the difference was statistically significant. Our present study achieved a similar predictive performance with these two studies. In our study, the AUC of our clinical model and the radiomics score were 0.767 (95%CI: 0.673–0.860) and 0.849 (95% CI: 0.783–0.914), respectively, which was similar with the results of the above two studies.

Deep learning has made significant progress in the field of medical image analysis by mining high-throughput information from medical images for recognition of images or prediction of gene expression [37–40]. Wang et al. developed an end-to-end deep learning model based on CT images, which obtained AUCs of 0.85 (95% CI 0.83–0.88) in the primary cohort and 0.81 (95% CI 0.79–0.83) in the independent validation cohort to predict EGFR mutation status in lung cancers, respectively [38]. Chen et al. developed a deep learning-based method for the automatic segmentation of meningiomas from multiparametric MR images, and the AUC of the radiomics model with automatic segmentation was comparable to the AUC of the manual segmentation model in the internal (0.95 vs. 0.93, *p* = 0.176) and external (0.88 vs. 0.91, *p* = 0.419) test cohort [37]. In this study, a pre-trained CNN, ResNet 50, was implemented to extract 2048 deep learning features from CT images of NSCLCs, and 15 features were found to be strongly associated with PD-L1 expression. Compared to the clinical model and radiomics model, the DLR-score demonstrated the highest predictive performance for PD-L1 expression with AUCs of 0.938(95%CI: 0.899–0.977) and 0.818(95%CI:0.727–0.910) in the training cohort and the validation cohort, respectively. The optimal cut-off value derived from DLR-score was 0.246 achieved sensitivity, specificity, PPV, NPV, and accuracy, of 90.6%, 87.8%, 64.4%, 97.5%, and 88.3%, respectively, which were more improved than the other two models. The decision curve analysis also showed that the clinical net benefit of the DLR-score was higher than that of clinical models and radiomics score, both in the training and validation cohort.

Several limitations of this study need to be acknowledged. First, this was a retrospective study with a small sample size and no external validation cohort. Second, the imbalance distribution between PD-L1 positive and negative expression may impact on the predictive performance of the model. Third, to avoid overfitting, transfer learning often requires a large sample size, and the sample size in this study was clearly not sufficient for 3D analysis, so we used the image of the largest tumor area, rather than using 3D whole tumor volume to extract DL features [17]. However, this approach is time-saving and may be more clinically appropriate. Finally, owing to surgical confirmed cases at an early stage and a short follow-up period, only several patients received ICIs treatment after surgery due to recurrence or metastasis at present. Therefore, the predictive performance of the CT-based deep learning radiomics signature for treatment response was not evaluated at this study.

## Conclusion

In conclusion, this study developed clinical, radiomics and deep learning models to predict PD-L1 expression in NSCLCs non-invasively. It showed the CT-based deep learning radiomics model could achieve clinically acceptable predictive performance in both training and validation cohorts. The deep learning radiomics signature could offer a surrogate imaging biomarker or a complement for IHC analysis, which could facilitate clinical decision support in identifying NSCLC patients who are likely to benefit from ICIs treatment.

## Data Availability

The datasets analyzed during the current study are available from the corresponding author on reasonable request.

## Abbreviations

3D-VOIs: Three-dimensional volume of interest
AUC: Area under the curve
CNN: Convolutional neural network
DICOM: Digital Imaging and Communications in Medicine
DL: Deep learning
DLR-score: Deep learning radiomics score
ICCs: Intraclass correlation coefficients
ICIs: Immune checkpoint inhibitors
IHC: Immunohistochemistry
LASSO: Least absolute shrinkage and selection operator
NSCLC: Non-small cell lung cancer
PACS: Picture archiving and communication system
PD-1: Programmed cell death protein 1
PD-L1: Programmed cell death ligand 1
Rad-score: Radiomics score
ROI: Regions of interest
TPS: Tumor proportion score
